# The Effects of Martial Arts Training on Attentional Networks in Typical Adults

**DOI:** 10.3389/fpsyg.2018.00080

**Published:** 2018-02-08

**Authors:** Ashleigh Johnstone, Paloma Marí-Beffa

**Affiliations:** School of Psychology, Bangor University, Bangor, United Kingdom

**Keywords:** Martial Arts, attention training, attention, cognitive control, typical adults, alerting, cross-sectional

## Abstract

There is substantial evidence that training in Martial Arts is associated with improvements in cognitive function in children; but little has been studied in healthy adults. Here, we studied the impact of extensive training in Martial Arts on cognitive control in adults. To do so, we used the Attention Network Test (ANT) to test two different groups of participants: with at least 2 years of Martial Arts experience, and with no experience with the sport. Participants were screened from a wider sample of over 500 participants who volunteered to participate. 48 participants were selected: 21 in the Martial Arts group (mean age = 19.68) and 27 in the Non-Martial Arts group (mean age = 19.63). The two groups were matched on a number of demographic variables that included Age and BMI, following the results of a previous pilot study where these factors were found to significantly impact the ANT measures. An effect of Martial Arts experience was found on the Alert network, but not the Orienting or Executive ones. More specifically, Martial Artists showed improved performance when alert had to be sustained endogenously, performing more like the control group when an exogenous cue was provided. This result was further confirmed by a negative correlation between number of years of Martial Arts experience and the costs due to the lack of an exogenous cue suggesting that the longer a person takes part in the sport, the better their endogenous alert is. Results are interpreted in the context of the impact of training a particular attentional state in specific neurocognitive pathways.

## Introduction

Being able to attentionally focus on a task, and therefore avoid distraction, is fundamental to achieving our goals. Despite its central role in human adaptation to life, it is one of the most vulnerable cognitive functions. This is evidenced by the level of research showing the number of variables that deficits in attention can be attributed to, such as genetics (Durston et al., 2006), mental illness (Clark et al., 2002), and traumatic brain injury (Shah et al., 2017), among others. Age has perhaps the biggest influence on attentional control with a large amount of research discussing the decline in this function in older adults (Milham et al., 2002; Kray et al., 2004; Jennings et al., 2007; Deary et al., 2009; Carriere et al., 2010; Dorbath et al., 2011). Deterioration of attentional control is variable but generally progressive, establishing it as the best predictor of cognitive dysfunction in older people. In neural terms, attentional control is achieved by the coordinated activation of a number of attentional networks with various specialities depending on the type of control required, although not all of these networks are affected by age in the same manner (Jennings et al., 2007).

Compared to how easily attentional control seemingly declines, little is known about whether we can enhance this function, and if so, how. In this paper, we evaluate the impact of Martial Arts experience on three different attentional networks: Alert, Orienting, and Executive. These networks have been neuroanatomically validated and reported as being largely independent of one another (Fan et al., 2002). The results provided in this paper are important in aiding understanding of the impact of experience on these networks, whilst also highlighting potential intervention strategies.

### Attentional Control in Martial Arts

Tang and Posner (2009) suggested that there are two different ways to improve attentional control: Attention Training (AT, also called Network Training; Voelker et al., 2017) and Attention State Training (AST). AT comes from Western cultures and is mostly based on specific task practice; over the past decade it has become popularized and marketed as ‘brain training’ games (Boot et al., 2008; Bavelier and Davidson, 2013). This means that much research into AT focuses on training participants on a certain task to improve a specific cognitive skill, yet these improvements often are not transferable to tasks measuring other skills (Rueda et al., 2005). For example, training at an attentional task will only improve the skills required for attentional tasks similar in nature (Thorell et al., 2009). Despite this, improvements are often found in this type of AT research. Participants given training in playing an action video game were shown to present an increase in visual attention, in comparison to those given training in playing Tetris (Green and Bavelier, 2003). This is possibly due to the need to stay vigilant whilst also scanning the screen for targets or enemies during this type of game. In addition to the improvement not being transferable, this improvement seems to be short-term, rather than the long-term improvement researchers are striving for Tang and Posner (2009).

On the other hand, AST is based on Eastern cultures and aims to improve attention through a change in state of mind and body, also claiming to provide a better transference to other tasks not specifically trained by the activity (Tang and Posner, 2009). AST can be found in activities such as yoga, mindfulness, meditation, and Martial Arts. Gothe et al. (2013) used healthy, adult participants to investigate the effects of yoga on cognitive control. Participants were asked to visit the laboratory on three occasions to complete some computerized behavioral tasks after a different activity on each day: (1) a 20-min yoga session; (2) a 20-min exercise routine on a treadmill; (3) no activity in order to collect baseline data. The order of the three activities was randomized. A flanker task and an n-back task were used to provide measures of attentional control, and results indicated that the yoga session provided an improvement across both of these tasks. Interestingly, these benefits were not seen after the aerobic exercise condition, perhaps suggesting that the exercise element of yoga is not the sole force behind the effects. Similarly, Moore and Malinowski (2009) found a correlation between mindfulness experience and improved performance in attention and response inhibition tasks. However, unlike the Gothe et al. (2013) experiment, this was a cross-sectional design using the amount of mindfulness experience as a variable rather than results after a single session.

Martial Arts includes similar aspects to mindfulness and yoga, and could potentially produce similar improvements in attentional control, although much of the research with Martial Arts has been conducted with school aged children (Diamond and Lee, 2011). For example, during an academic year, an average of three sessions of Taekwondo per week showed improvements in working memory and attention, as well as parentally-reported benefits in concentration and behavioral inhibition (Lakes et al., 2013). Additionally, a recent large-scale review of 84 studies conducted by Diamond and Ling (2016) found that Martial Arts, mindfulness, and Montessori Teaching produced the widest range of benefits in executive control tasks in children when compared with other interventions such as team sports, aerobic exercises, board games, or adaptations to the school curriculum. This review also raised an important point, noting that the greatest benefits were found in the children with the lowest starting scores in cognitive tests, and those from lower socio-economic backgrounds. This observation indicates that the greatest benefits from this type of intervention should be observed in those who display poor cognitive control and that neurotypical populations composed of developed young adults may already be at a ceiling in their attentional performance. Indeed, reports of improved cognitive abilities in younger adults are rare. Most of the benefits have been found in the sensorymotor system, involving corticospinal excitability due to long term training in Karate (Moscatelli et al., 2016b), or in the excitability of the motor cortex in Taekwondo athletes (Moscatelli et al., 2016c). Interestingly, some of these pathways coexist with more cognitive networks, such as attentional networks (as reviewed further on), raising the possibility of successfully finding changes in cognition with neurotypical adults despite the lack of previous reports.

Conversely, one would expect some improvements in older adults due to the evidence suggesting an age-related decline in cognitive control. Kray et al. (2004) suggested that if cognitive control was plotted on a graph along the lifespan, then it would take the shape of an inverted ‘U,’ with performance improving as a person ages, remaining relatively stable during early adulthood, and then declining again as a person grows older. Studies using older populations to investigate the effects of Martial Arts on attentional control remain elusive, possibly due to the physical demands the sport requires, however that is not to say that this type of research is impossible. Jansen and Dahmen-Zimmer (2012) recruited participants aged 67–93 to compare the effects of Karate training in comparison to general physical exercise training, and cognitive training. This training took place over 20 sessions over 3–6 months, yet despite an increase in well-being reported by those in the Karate training group, there were no significant effects on cognitive speed or working memory across any of the groups.

However, it is important to note that outside laboratories, Martial Artists usually measure their differences in training in terms of years, rather than weeks or months, so it is conceivable that short interventions would not achieve the state of mind characteristic of the discipline. Witte et al. (2015) built on previous research and studied three different groups of participants, with an age range of 63–83. They compared a group training in Karate, with another training in fitness and with a passive control group that did not complete any sports intervention. The results showed that the Karate group displayed small improvements in each of the four tasks performed. In a test of divided attention, for example, this improvement was not quite significant (*p* = 0.063) after 5 months but, after another 5 months of extra training, the level of improvement further increased, reaching more reliable effects (*p* = 0.002). These results clearly suggest that, at least in adults, potential benefits may need a longer time of training to emerge than those normally used in pre–post intervention studies.

To sum up, much of the research into the effects of Martial Arts on attentional and cognitive control has used either school-aged children or older adults. There appears to be a lack research focusing on healthy, neurotypical, adult participants. This population seems to need longer periods of training to show any improvement in other transference tasks, and this is the gap that we aim to fill with our current research.

### The Attention Network Test

A limitation of comparing much of the previous research is the wide range of measures used to assess attentional control, potentially leading to inconsistency across studies. One way to reduce this problem is to avoid using general measures (such as academic results or IQ) that result in difficulties isolating the core mechanisms behind the benefits. The problem could also be countered by using tasks which have been validated as measuring specific functions that have been localized to neuroanatomical locations.

Petersen and Posner (2012) discussed recent literature in attentional control and confirmed the existence of three core networks of attention in the human brain: Alert, Orienting, and Executive (Posner and Petersen, 1990). They suggested that these networks are independent of each other, each having distinct neuroanatomical structures, and responsible for a different aspect of attention. The alert index is related to optimal vigilance, Orienting has associations with the spatial location of targets, and executive has been linked with conflict resolution. Measures of these indexes can be collected using the Attention Network Test (ANT; Fan et al., 2002), which utilizes a modified flanker task with four cues types to produce various trial types. The Alert index gives a measure of how well a person is able to respond to targets appearing at unpredictable intervals (uncued) compared to a predictable one (time cued). The Orienting index assesses how well-participants can orient to a target that appears in an unpredictable location (uncued) compared to a certain one (spatially cued). Finally, the executive index evaluates how well-participants can resolve response conflict in a flanker task, where distractors evoke the same response as the target (congruent) or the opposite one (incongruent). Behaviorally, all these three indexes are interpreted as costs, where large differences in RTs or accuracy reflect poor control (Fan et al., 2002; Jennings et al., 2007; Petersen and Posner, 2012).

Functional magnetic resonance imaging (fMRI) has been used to assess the neural activity related to the three attentional networks measured by the ANT. It has been suggested that these three networks are independent of each other, and while there is some overlap, the functional response for each network has a distinct anatomical location (Fan et al., 2005). The Alert index seems to involve norepinephrine circuits connecting the *locus coruleus* with the right frontal and parietal cortices. The Orienting index is mostly driven by acetylcholine areas engaging the superior parietal cortex, temporoparietal junction frontal eye fields and superior colliculus. Finally, the Executive network activates dopamine based areas including the anterior cingulate, lateral and ventral prefrontal cortices, and the basal ganglia. When a particular sensory event is presented, it is believed that the coordinated activation of these three networks makes it possible to react to them with fast and accurate responses.

Training in Martial Arts is a wide-reaching experience involving not only a great level of motor training but also a mental state of concentration and reactivity to targets with a strong social context. Because of this, it is difficult to confidently predict where the improvements, if any, should be observed. There are, however, different aspects of the training that could impact directly these indexes. For example, during sparring, Martial Artists need to continuously scan the body of the opponent for an opening where they can score. As this may happen at any particular time, training in sparring may transfer to other tests involving target detection at random intervals, as measured by the Alert index. In addition to scoring, the Martial Artist needs to avoid and block any incoming hit from the opponent. This requires not only good timing (also linked to the alert system) but enhanced spatial orienting to the exact location where the hit comes from. Following the example of sparring, Martial Artists also throw feigned punches and kicks to distract the opponent’s attention in order to score with an unexpected move. Not reacting to these in order to better respond to the real ones should require response conflict control of the type measured by the Executive index. Of course, it is not just sparring that is involved in Martial Arts training. Equally, these aspects are not exclusive of Martial Arts and can be shared with many other activities such as tennis, fencing, dancing, etc. But they at least represent a context of repetitive training on specific skills that are comparable to those used in AT studies, such as brain training. With the added element of concentration, meditation, and discipline (as is typical in AST research), it provides a promising strategy for training in attentional control.

In this study, we compared two groups of participants screened from a wider sample of 500 young adults. One group was composed of Martial Artists with at least 2 years of experience while the others had no previous experience with Martial Arts. Because of the requirements of extensive training, assignation to the groups could not be random, so special care was taken during the matching process to eliminate the most relevant potential confounds. As there is no previous literature of the influence of different demographics on ANT, we previously ran a pilot where these confounds were detected.

The aim of this study was to assess the performance of Martial Artists and Non-Martial Artists on the three indexes of attention, as measured by the ANT. We hypothesized that smaller indexes, reflecting improved performance, would be observed in the Martial Arts group in comparison to Non-Martial Artists.

## Materials and Methods

### Participants and Screening Procedure

An unpublished pilot study using an unbiased random sample of 41 undergraduate students of Psychology at Bangor University was used to test the ANT in the general population according to different demographic and lifestyle factors. This pilot showed that Age and Body Mass Index (BMI) both had significant effects on ANT performance, and so based on this we decided to match the Martial Arts and Non-Martial Arts groups mainly on these two variables among others.

Using G^∗^Power 3.0.10, an *a priori* calculation of optimal sample size was calculated based on parameters taken from the pilot study. When using stringent criteria such as a correlation of 0.6 between measures, a desired power of 0.95, and an alpha level set to 0.05, it was estimated that a minimum sample size of 30 participants would be needed to reach an effect size of 0.25 from the required 2x2x2 ANOVA (see section “Design and Procedure”). This would result in 15 participants in each of the two participant groups.

A screening questionnaire was introduced and distributed online to over 500 new people including Bangor University undergraduates and non-students from the local community. These responses then made up a participant pool which was used to create two experimental groups matched on the aforementioned variables: one group with no Martial Arts experience (*n* = 27, five males), and the second with those who had undertaken Martial Arts practice during the last 2 years (*n* = 21, six males). The Martial Arts group was made up of participants with experience in Karate (5), Taekwondo (3), Kickboxing (3), Jujitsu (3), Tai Chi (2), Judo (2), Thai Boxing (2), and Kung Fu (1). This sample exceeded the minimum sample size estimated by the power calculation. The participants from these groups were invited to participate in the ANT phase. The Non-Martial Arts group reported taking part in activities such as going to the gym, playing team sports, meditating, praying, and playing musical instruments.

Students from Bangor University were reimbursed for their time with course credits, and those from the community were given a monetary token of £6. All participants were neurotypical, had normal or corrected to normal vision, and normal hearing. This study gained approval from the Bangor University Ethics and Governance Committee (Ethics Approval #2015-15553) and have been performed in accordance with the ethical standards laid down in the 1964 Declaration of Helsinki and its later amendments. As a condition of this approval, all participants provided fully informed consent prior to taking part. Information regarding the demographics of the selected participants can be found in **Table 1**.

**Table 1:** Descriptive data for key participant demographics.

	Martial Artists	Non-Martial Artists	*p*
*N* (male)	21 (6)	27 (5)	0.498
Age	19.68 ± 1.95	19.63 ± 1.11	0.905
BMI	22.50 ± 4.08	22.55 ± 1.91	0.961
Alcohol (units per week)	3.47 ± 5.26	4.59 ± 5.98	0.515
Other activities (hours per week)	4.11 ± 3.93	3.93 ± 2.73	0.856
Smokers	3	0	0.083
On medication	1	3	0.499
On a special diet	2	4	0.652
Health condition	3	3	0.645

*Values represent mean averages, ± standard deviation, or frequencies. p-Values show exact probability values. N represents number of participants with the number of males in parentheses.*

### Stimuli and Apparatus

**FIGURE 1:**
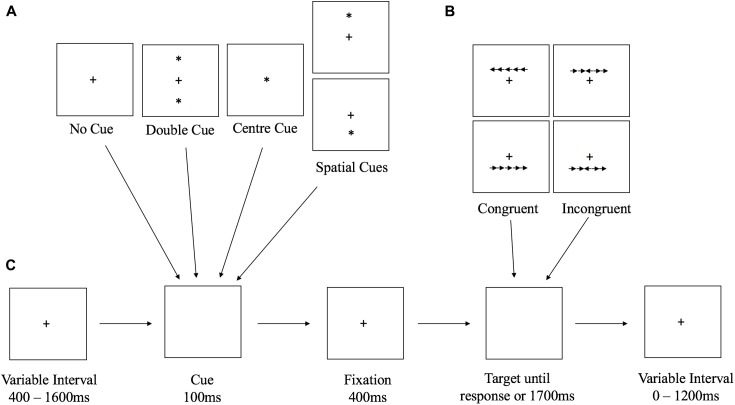
Diagram showing **(A)** all possible cue types, **(B)** the target types, and **(C)** trial timings and procedure.

This experiment was presented using EPrime 2.0 [Psychology Software Tools (PST)]. Responses were recorded using a QWERTY keyboard, with the ‘C’ and ‘M’ keys as the response keys. Target stimuli consisted of a row of five black arrows on a white background, facing to either the left or right side of the screen; each arrow subtended 0.53° of a visual angle, with a gap of 0.09°. The complete series of arrows subtended 2.73°. Participants were required to press the left key (C) or the right key (M) in response to the direction of the central arrow. This could be in either a congruent position (facing the same way) to the other arrows, or in an incongruent one (facing the opposite way). These were displayed either 0.71° of a visual angle above or below a fixation cross in the center of the screen (see **Figure 1**).

The target stimulus was preceded by one of four cue configurations (**Figure 1**): no cue, center cue, double cue, and spatial cue. Each cue was made up of a black asterisk the same size as the fixation cross (0.44° of a visual angle tall; 0.44° wide) and appeared for 100 ms before the target (**Figure 1**). During the no cue condition, an asterisk did not appear, instead, the fixation cross remained on screen. For the center cue conditions, the asterisk simply replaced the fixation cross. Double cue conditions consisted of an asterisk appearing both above and below the fixation cross. Finally, during spatial cue conditions, the asterisk appeared either above or below the fixation cross, and always provided a true indication of the location in which the target would appear.

Each trial began with a fixation cross presented during variable intervals (400–1600 ms) and ended with another fixation cross appearing just after the response to the target also with a variable duration to make the total interval time 1600 ms per trial. After the first interval, the cue appeared on screen for 100 ms, followed by another fixation cross for a fixed duration of 400 ms. The target then appeared and remained on screen until the participant responded, or until 1700 ms had passed. Responses exceeding this limit were recorded as errors.

### Design and Procedure

The study took a 2 (participant group) × 2 (trial type) × 2 design (target congruency – executive) design. For the Alert index this would look like 2 (Martial Arts vs. Non-Martial Arts) × 2 (no cue vs. double cue) × 2 (congruent target vs. incongruent target). Whereas for Orienting it would take the form of 2 (Martial Arts vs. Non-Martial Arts) × 2 (center cue vs. spatial cues) × 2 (congruent target vs. incongruent target). The Alert and Orienting networks come from cue manipulations, and are independent due to them using different trial types in their calculations, however the Executive network comes from a target manipulation and is therefore not independent of the Alert and Orienting networks. As a result, we will analyze this as an interaction.

Upon arrival in the laboratory, participants were provided with information about the experiment, given the opportunity to ask questions, and provided with a consent form. After receiving fully informed consent, participants were presented with the demographics questionnaire, before being asked to complete the ANT by responding to the direction of a central arrow as described earlier.

A practice block of 24 trials was presented to participants to ensure all instructions were understood; no feedback was provided. Once completed, participants moved onto the experimental block of 128 trials, before having a break of a length determined by the participant, which was then followed by another 128 trials. Again, no feedback was provided.

### Data Analysis

All data was pre-processed within EPrime 2.0 (PST). Incorrect trials were removed from the analysis, as were those with a response time greater than 1000 ms. Once the filtering in EPrime was complete, the data was moved over to SPSS v.22 for statistical analysis, and split into the two participant groups based on the criteria mentioned above for Martial Arts experience. The significance level was set at *p* ≤ 0.05. Descriptive statistics are presented as mean averages, ± standard deviation for continuous variables, and frequencies for categorical variables (see **Table 1**). Differences between the groups were estimated using independent samples *t*-tests, whilst differences in frequencies were assessed using chi square. The three indexes, Alert, Orienting, and Executive, were then created using the calculations described by Fan et al. (2002). These are expressed as mean cost indexes. Mean RT averages per participant, per condition were analyzed through three different general linear models (see **Table 2**). Effect sizes for these effects are estimated through partial eta squared. When Martial Arts group differences were found, correlations were conducted with years of experience using the Pearson’s coefficient.

**Table 2:** *F*-values, probability values (*p*) and effect sizes ($\eta_{p}^{2}$), for all conducted general linear models.

	*F*	*p*	$\eta_{p}^{2}$
**Executive**
Executive	113.92	0.00^∗∗∗^	0.71
Group	1.34	0.25	0.03
Executive × Group	0.02	0.90	0.00
**Executive vs. Alert**
Executive	52.60	0.00^∗∗∗^	0.53
Group	1.05	0.31	0.02
Alert	71.30	0.00^∗∗∗^	0.61
Alert × Group	5.64	0.02^∗∗^	0.11
Executive × Group	0.27	0.61	0.01
Alert × Executive	4.18	0.05^∗^	0.09
Three-way interaction	0.34	0.57	0.01
**Executive vs. Orienting**
Executive	160.93	0.00^∗∗∗^	0.78
Group	1.58	0.22	0.03
Orienting	111.14	0.00^∗∗∗^	0.71
Orienting × Group	0.71	0.41	0.02
Executive × Group	0.26	0.61	0.01
Orienting × Executive	10.52	0.00^∗∗^	0.19
Three-way interaction	3.09	0.09	0.06

*^∗^p ≤ 0.05; ^∗∗^p ≤ 0.01; ^∗∗∗^p ≤ 0.001*.

## Results

Data were separately analyzed for each of the attentional indexes.

### Executive

**FIGURE 2:**
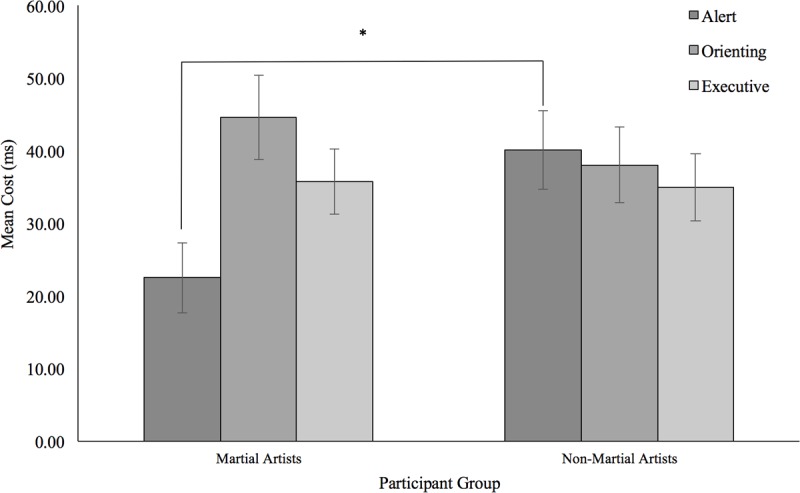
Graph depicting the mean cost for each of the three attentional network, for both participant groups. Error bars represent Standard Error. ^∗^*p* < 0.05.

When the Executive index was analyzed in isolation, we found an overall increase of 36 ms for incongruent trials compared to congruent ones [*F*(1,46) = 1013.92; *p* < 0.001; $\eta_{p}^{2}$ = 0.1]. This effect was almost identical for the Martial Arts group (36 ms) compared to the Non-Martial Arts one (35 ms, *F* < 1) (see **Figure 2**).

### Executive vs. Alert

**FIGURE 3:**
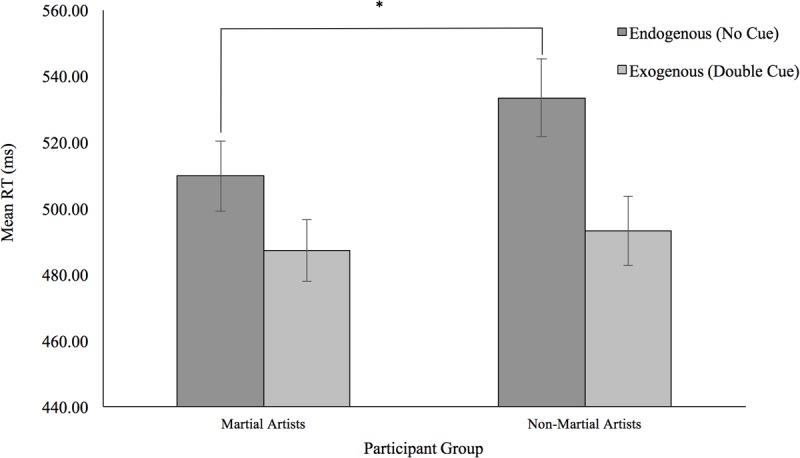
Graph depicting the mean RT for the trial types that make up the Alert index, no cue trials and double cue trials. Mean RTs are displayed for both participant groups. Error bars represent Standard Error. ^∗^*p* < 0.05.

Mean RTs per participant per condition were submitted to a mixed factor analysis of variance (ANOVA) with the Martial/Non-Martial Arts variable as a grouping factor and the Type of Cue (Double Cue, no Cue) and Congruency (Congruent, Incongruent) as repeated measures. Results indicated no overall differences in RTs across the groups (*p* = 0.31). Responses to targets preceded by the double cue were 32 ms faster than those without a cue as would be expected as a measure of Alert. More importantly, this benefit from the double cue was 18 ms smaller in the Martial Arts group compared to the Non-Martial arts group [*F*(1,46) = 5.64; *p* = 0.022; $\eta_{p}^{2}$ = 0.642] (see **Figure 2**). Although group differences did not reach significance in any of the conditions, Non-Martial Artists were found to be particularly slower than the Martial Artists when no cue was presented (24 ms), while both groups seemed more similar with the double cue (6 ms) (see **Figure 3**). Congruent trials were overall 32 ms faster than incongruent ones [*F*(1,46) = 52.59; *p* < 0.001; $\eta_{p}^{2}$ = 0.1], but this effect did not change with the group (*F* < 1). Interestingly, the executive congruency effects were more evident in the double cue trials (38 ms) than with no cue (27 ms) [*F*(1,46) = 4.18; *p* = 0.047; $\eta_{p}^{2}$ = 0.516]; but this was found in general for all participants and did not change across the groups (*p* = 0.57).

### Executive vs. Orienting

Mean RTs per participant per condition were also submitted to a mixed factor analysis of variance (ANOVA) with the Martial/Non-Martial Arts variable as a grouping factor and the Type of Cue (Spatial, Center Cue) and Congruency (Congruent, Incongruent) as repeated measures. As before, no overall differences were found between the Martial Arts and the Non-Martial Arts groups (*p* = 0.22) (see **Figure 2**). Congruent trials were found to be 38 ms faster than the incongruent ones [*F*(1,46) = 160.93; *p* < 0.0001; $\eta_{p}^{2}$ = 1]. Also, spatial cues produced responses that were 41 ms faster than the single central cue [*F*(1,46) = 111.14; *p* < 0.0001; $\eta_{p}^{2}$ = 1]. Interestingly, the congruency effects changed depending on the type of spatial cueing [*F*(1,46) = 10.52; *p* = 0.002; $\eta_{p}^{2}$ = 0.89], with a flanker congruency effect of 47 ms in the center cue condition congruent trials that was reduced to 29 ms with the spatial cue.

### Attentional Networks Correlations

We analyzed whether there were any interactions across attentional networks by calculating the correlations across the three indexes. Results demonstrated a marginally significant correlation between the Alert and Executive indexes only (*r* = -0.262; *p* = 0.072).

A correlation was also done on the three indexes of attention and the number of years of Martial Arts practice. No significant correlation between Orienting and number of years was found, *r* = 0.121, *n* = 46, *p* = 0.421, nor between Executive and number of years, *r* = 0.039, *n* = 46, *p* = 0.798. However, a correlation nearing significance was found between the Alert index and the number of years of practice, *r* = -0.274, *n* = 46, *p* = 0.065.

### Accuracy

Finally, a series of analyses were run on the accuracy of responses to each trial type. For each participant, a percentage (%) accuracy score was calculated for each type of trial (cue type and congruency type), and these were then compared between groups. There were no significant differences between the two participant groups for any trial type for the ANT, suggesting that all trials were equally as difficult. Less than 16% of overall responses were recorded as errors.

## Discussion

In this paper, we provide evidence that training in Martial Arts is associated with improvements in the Alert attentional network. This appears to be a specific benefit that boosts endogenous preparation for uncertain targets, as suggested by the increased benefits in the uncued conditions in comparison to a lack of improvement in the cued conditions. This means that when an upcoming target had no cue, the Martial Artists performed at a higher level, however when the target had a reliable cue, these group differences disappeared. Importantly, the Alert benefits observed in the MA group was further supported by the negative correlation found between the Alert index and the number of years of training.

The use of ANT allows us to speculate on the nature of these benefits, as explained in the introduction. Previous research with this task using neuroimaging techniques found that the Alert index is linked to the activation of a norepinephrine based network connecting the *locus coeruleus* with the right frontal and parietal cortices, as well as the anterior cingulate cortex (ACC) and orbitofrontal cortex (OFC; Nieuwenhuis et al., 2005; Raz and Buhle, 2006; Petersen and Posner, 2012). The *locus coeruleus* is a nucleus in the brainstem in charge of producing norepinephrine, which has an excitatory effect on the rest of the brain, resulting in an increased level of arousal. As a result of this activation, different parts of the brain involved in perceptual and motor processing get primed to enable faster responses to stimuli (Moscatelli et al., 2016a,b; Monda et al., 2017).

It is not yet clear which aspect of Martial Arts training may be driving the effect on the alert index, or indeed where the effect is coming from. Further work using neuroimaging techniques may allow us to gain an insight into these details. For example, Fan and Posner (2004) suggested the use of diffusion tensor imaging (DTI) to look at the attentional networks’ functional connectivity; by understanding how these circuits work in a typical group of participants, we could then begin to investigate whether Martial Arts experience has any effect, which would then be able to show us where the effect on alert is observed at a neural level. Of course, it is conceivable that Martial Artists who trained for years on fast reactions to stimuli may have modeled their brains to lower the activation threshold of areas involved in perceptual processing and motor control (Moscatelli et al., 2016c). However, we would expect this influence to appear across all conditions, for both predictable and unpredictable targets, simply inducing faster reaction times, rather than any exclusive benefits. This idea is not supported by the results which suggested no significant differences in overall RTs in the Martial Arts group in comparison to the Non-Martial Arts controls.

An interesting aspect of our results is that the strongest benefits seem to appear more specifically in the unpredictable condition. Somehow, our Martial Artists seem to be more capable of inducing these increases in arousal to improve sensorimotor processing endogenously without the aid of external cues. Indeed, there is evidence that endogenous time allocation of attention to the particular moment when a target appears improves identification of masked targets that otherwise would have been unconsciously processed (Naccache et al., 2002). More importantly, it has also been found that identifications were better for targets closer to the expected time frame than for more distant ones in time. When this is considered in relation to our findings, it raises the possibility that Martial Artists may endogenously hold the level of vigilance for longer periods of time reaching the unpredictable target in a more efficient way than controls.

This interpretation is supported by recent findings of increased excitability of the corticospinal motor system in Karate athletes (Moscatelli et al., 2016a,b; Monda et al., 2017). In this study, the authors found that this greater excitability from the Karate group was evidenced in faster reaction times to targets appearing in variable intervals (as is standard in the Reaction Time [RTI] test from the Cambridge Neuropsychological Test Automated Battery [CANTAB^®^]). Related findings from this team have also found excitability of the motor cortex in Taekwondo athletes (Moscatelli et al., 2016c), suggesting that this effect may be found in other types of Martial Arts. In the current study, when the target appeared in a predictable interval, no group differences were found. Our Martial Arts group were faster than the Non-Martial Arts group only with unpredictable targets, thereby supporting Moscatelli colleagues findings. Interestingly this RTI task was also described as a vigilance task. Our results can be seen as a step forward, further suggesting that the excitability of this corticospinal motor system may be linked to the improved activity in the Alert network due to Martial Arts practice.

Although previous research seems to assume that the three attentional networks studied here largely independent, there is some evidence of influences across them. For example, spatial orienting seems to have a fundamental role in the activation of competing responses during a flanker task in what it would seem like a modulation of the Orienting network over the Executive one (Vivas and Fuentes, 2001). Also, when testing neglect patients, increased alert can be used to improve target detection in the hemifield contralateral to the site of the lesion (Robertson et al., 1995), demonstrating an influence of the Alert network over the Orienting one. In our data, we did not find any correlation between Alert and Orienting, neither Orienting with Executive. However, we did find a strong correlation between Alert and Executive, since greater congruency effects were found in the predictable condition. Further support may come from studies finding that increases in norepinephrine improve executive response selection (Chamberlain et al., 2006). This also fits well with results described earlier in which response congruency effects were only found at the spatially cued location (Vivas and Fuentes, 2001). Basically, the executive resolution of conflict elicit by incongruent flankers requires first the selection of the target, both spatially and temporally. Although this is an interesting aspect of the data, it nevertheless did not change with the group, not being affected by training in Martial Arts.

The benefits associated with MA training in our study seem to be exclusive to the Alert system, mostly with regards to endogenous alert. Importantly, this improvement increases with years of practice extending up to 18 years. These results are important because it highlights the potential difficulties of getting significant results from studies of using randomized groups with a training intervention of only a few months. Nevertheless, one of the biggest disadvantages of using cross-sectional samples is the lack of control of group variables. In order to improve the control over the current study, participants were carefully matched on various variables. To find two homogeneous participant groups, demographic information for over 500 people was collected and then filtered based on age, BMI, lifestyle, and health factors such as smoking status, and level of education. To avoid ending up with an active participant group and a passive participant group, we ensured that control participants were only recruited if they reported taking part in several hours of activity per week. The activities reported included gym time, football, and basketball among others, suggesting that the control participants were just as active as the Martial Artists. We believe that this is an important variable to use with regards to matching the groups to ensure a similar level of fitness due to previous research suggesting a link between fitness and cognitive control. Our estimation, however, has been based on a non-validated self-report measure so should be considered with caution. In any case, the descriptive data showed no significant differences in terms of hours of participating in other activities per week between the two groups.

A further consideration comes from the heterogeneity of participants in the Martial Arts group, specifically in relation to the styles of Martial Arts. In the current paper, different styles of Martial Arts with variations in both training and philosophy are used. This was done under the assumption that all of them would contain elements of physical and mental training in line with Tang and Posner (2009) AST classification, found to cause improvements in executive control (Moore and Malinowski, 2009; Gothe et al., 2013). However, Weiser et al. (1995) suggested that Martial Arts exist on a continuum with more meditative styles on one end, and more combative styles on the other. This suggests that it may be important to consider potential differences in style in various forms of Martial Arts. Further research intends to assess these possible differences, in the hope that it could lead to a greater understanding of the underlying drivers behind the cognitive improvements associated with Martial Arts.

The current research suggests that the alert network of attention differs between people with Martial Arts experience and those without this experience. Whilst this effect was only found in one of the three known attentional networks, it supports previous work which suggests changes in control as a result of taking part in Martial Arts, whilst also extending the research field into populations of neurotypical adults. Further research should seek to replicate this finding, and discover the underlying reasons for the effect solely appearing in the Alert network and not Orienting or Executive. This may help improve our understanding of how activity in different attentional networks can be ‘trainable’ or able to be improved through Martial Arts.

## Data Availability

The datasets analyzed during the current study are available from the corresponding author on reasonable request.

## Author Contributions

AJ and PM-B equally contributed to the development, data collection, analysis and interpretation, and manuscript write- up of this research.

## Conflict of Interest Statement

The authors declare that the research was conducted in the absence of any commercial or financial relationships that could be construed as a potential conflict of interest. The reviewer DG and handling Editor declared their shared affiliation.
